# Preferential uptake of SARS-CoV-2 by pericytes potentiates vascular damage and permeability in an organoid model of the microvasculature

**DOI:** 10.1093/cvr/cvac097

**Published:** 2022-06-16

**Authors:** Abdullah O Khan, Jasmeet S Reyat, Harriet Hill, Joshua H Bourne, Martina Colicchia, Maddy L Newby, Joel D Allen, Max Crispin, Esther Youd, Paul G Murray, Graham Taylor, Zania Stamataki, Alex G Richter, Adam F Cunningham, Matthew Pugh, Julie Rayes

**Affiliations:** Institute of Cardiovascular Sciences, College of Medical and Dental Sciences, University of Birmingham, Vincent Drive, B15 2TT, Birmingham, U.K; Institute of Cardiovascular Sciences, College of Medical and Dental Sciences, University of Birmingham, Vincent Drive, B15 2TT, Birmingham, U.K; Institute of Immunology and Immunotherapy, University of Birmingham, Birmingham, B15 2TT, U.K; Institute of Cardiovascular Sciences, College of Medical and Dental Sciences, University of Birmingham, Vincent Drive, B15 2TT, Birmingham, U.K; Institute of Cardiovascular Sciences, College of Medical and Dental Sciences, University of Birmingham, Vincent Drive, B15 2TT, Birmingham, U.K; School of Biological Sciences, University of Southampton, Southampton SO17 1BJ, U.K; School of Biological Sciences, University of Southampton, Southampton SO17 1BJ, U.K; School of Biological Sciences, University of Southampton, Southampton SO17 1BJ, U.K; Forensic Medicine and Science, University of Glasgow, Glasgow, UK; Institute of Immunology and Immunotherapy, University of Birmingham, Birmingham, B15 2TT, U.K; Health Research Institute, University of Limerick, Limerick, Ireland; Institute of Immunology and Immunotherapy, University of Birmingham, Birmingham, B15 2TT, U.K; Institute of Immunology and Immunotherapy, University of Birmingham, Birmingham, B15 2TT, U.K; Institute of Immunology and Immunotherapy, University of Birmingham, Birmingham, B15 2TT, U.K; Institute of Immunology and Immunotherapy, University of Birmingham, Birmingham, B15 2TT, U.K; Institute of Immunology and Immunotherapy, University of Birmingham, Birmingham, B15 2TT, U.K; Institute of Cardiovascular Sciences, College of Medical and Dental Sciences, University of Birmingham, Vincent Drive, B15 2TT, Birmingham, U.K

**Keywords:** SARS-CoV-2, COVID-19, Endothelial permeability, Thrombosis, Organoids, Vasculopathy

## Abstract

**Aims:**

Thrombotic complications and vasculopathy have been extensively associated with severe COVID-19 infection, however the mechanisms inducing endotheliitis and the disruption of endothelial integrity in the microcirculation are poorly understood. We hypothesized that within the vessel wall, pericytes preferentially take up viral particles and mediate the subsequent loss of vascular integrity.

**Methods and Results:**

Immunofluorescence of post-mortem patient sections were used to assess pathophysiological aspects of COVID19 infection. The effects of COVID-19 on the microvasculature were assessed using a vascular organoid model exposed to live viral particles or recombinant viral antigens. We find increased expression of the viral entry receptor ACE2 on pericytes when compared to vascular endothelium, and a reduction in the expression of the junctional protein CD144, as well as increased cell death, upon treatment with both live virus and/or viral antigens. We observe a dysregulation of genes implicated in vascular permeability including NOTCH3, angiopoietin-2 and TEK. Activation of vascular organoids with IL-1β did not have an additive effect on vascular permeability. Spike antigen was detected in some patients’ lung pericytes, which was associated with a decrease in CD144 expression and increased platelet recruitment and VWF deposition in the capillaries of these patients, with thrombi in large vessels rich in VWF and fibrin.

**Conclusions:**

Together our data indicates that direct viral exposure to the microvasculature modelled by organoid infection and viral antigen treatment result in pericyte infection, detachment, damage and cell death, disrupting pericyte-endothelial cell crosstalk and increasing microvascular endothelial permeability, which can promote thrombotic and bleeding complications in the microcirculation.

**Translational Perspective:**

Endotheliitis is a serious complication of severe COVID-19 patients which remains poorly understood. We identify a pericyte mediated mechanism by which the vasculature becomes compromised, contributing to thrombotic complications, highlighting important avenues for the development of therapies.

**Graphical Abstract**

## Introduction

Severe acute respiratory syndrome coronavirus 2 (SARS-CoV-2)-associated vasculopathy and endotheliitis are commonly observed in patients with severe COVID-19 [1]. The incidence of macrovascular thrombosis is relatively high in these patients, with arterial thrombosis and venous thromboembolism observed in over 30% of patients [2]. Endothelial activation and injury, disruption of cell membrane integrity, intussusceptive angiogenesis, and widespread microthrombi in alveolar capillaries have also been detected in lung autopsies [3-5]. Under basal conditions, microvascular endothelial cells (ECs) are tightly associated with specialized mural cells (pericytes) that act as integral anchors supporting endothelial vascular integrity and permeability [6]. Infection and subsequent cell damage induce mural cell activation and apoptosis, driving inflammation and disrupting interactions between pericytes and ECs, increasing vessel instability, vascular permeability and potentially driving thrombosis and bleeding [7]. Whether SARS-CoV-2 directly infects endothelial cells and contributes to cell damage and activation remains controversial [8,9,10], with increasing evidence supporting key roles for the stroma, as well as innate immune cell and platelet activation in endotheliopathy [11, 12]. Despite the prevalence of these pathologies, the mechanisms driving SARS-CoV-2-mediated endotheliitis and thrombosis both in large vessels and in capillaries remains poorly understood [5, 13, 14].

Following SARS-CoV-2 infection, binding of the viral spike glycoprotein to the host angiotensin-converting enzyme 2 (ACE2) promotes cellular entry, with different proteolytic reactions mediated by Transmembrane Serine Protease 2 (TMPRSS2) and co-receptors required for efficient virion infection [15, 16]. Infection-mediated disruption of the epithelial barrier allows the virus to infect the vessel wall, with the efficiency of infection being regulated by ACE2 and its co-receptors expression. ACE2 expression on human endothelium has been shown to be insufficient for viral replication and endothelial activation [8]. Furthermore, inoculation of primary lung endothelial cells with SARS-CoV-2 results in limited infection rates [17, 18], while spike pseudovirus can induce mitochondrial damage in endothelial cells [10]. In the absence of clear evidence showing endothelial infection by SARS-CoV-2, other cellular constituents of the vessel wall, such as pericytes, have emerged as potential target cells and sites of infection [19-23]. Indeed, ACE2 expression on brain and cardiac pericytes has been shown to support SARS-CoV-2 infection [22, 23]. Conversely, lung pericyte infection by SARS-CoV-2 and its effect on endothelial cell activation and integrity in particular, is not well studied [20-24]. This is at least partially due to the difficulty in modelling pericytes environment *in vitro*, a technical challenge which has been addressed in recent years with the development of organoid models. Indeed, microvascular healthy pericytes express ACE2 [15, 25, 26] and this expression can be further increased in pathological conditions such as lung fibrosis [27], a clinical complication observed in patient cohorts known to be vulnerable to COVID-19 infection[28]. The ability of SARS-CoV-2 to infect target cells is heavily dependent on the restricted expression of ACE2, TMPRSS2 and novel proteases and cofactors [19, 26]. Using vascular and kidney organoid models, *Monteil et al* showed that SARS-CoV-2 can directly infect vascular organoids, which can be reversed by human recombinant soluble ACE-2 [29]. However, the cell type involved in SARS-CoV-2 uptake and its effect on vessel wall cell activation and survival is not known.

In this study, we assessed the presence of the viral spike glycoprotein in pericytes in lung autopsies from eight patients with COVID-19. We reasoned that direct infection of pericytes with SARS-CoV-2 drives endothelial dysfunction and potentiates thrombotic complications in the microcirculation. We show a heterogenous presence of the spike glycoprotein in lung microvascular pericytes, in particular in patients with documented microvascular and macrovascular thrombosis. Infection of 3D vascular organoids mimicking the microvasculature with SARS-CoV-2, spike glycoprotein and nucleocapsid protein induced pericyte and endothelial death and altered endothelial permeability, independently of endothelial activation. We observed a preferential uptake of SARS-CoV-2 by pericytes associated with a dysregulation of genes regulating endothelial permeability such as NOTCH3 and angiopoietin-2. Our data suggest that pericytes are a critical site of SARS-CoV-2 infection and disruption of pericyte-endothelial interactions promotes thrombosis.

## Materials and Methods

### Ethical approvals

Collection of post-mortem FFPE tissue was approved (IRAS: 197937) for tissue obtained via prospective consent post-mortem and retrospective acquisition of tissue in which consent for use in research had already been obtained. Ethics for patient tissue were approved by the Health Research Authority (HRA) with an NHS (National Health Service) REC (Research Ethics Committee); approval issued by North East- Newcastle and North Tyneside 1 (19/NE/0336). All necessary patient/participant written consent has been obtained and the appropriate institutional forms have been archived. This research adheres to the tenets of the Declaration of Helsinki.

**Patients**

*Post mortem* formalin-fixed and paraffin-embedded lung sections from eight COVID-19, one MERS-CoV, one rhinovirus and two control (non-respiratory-associated diseases) patients. COVID-19 samples were from pre-hospital and hospital deceased patients, with time from symptoms to death ranging from 0-36 days, and ages ranging from 59-89 years old. Patients were not on mechanical ventilation and all had comorbidities (highlighted in Supplementary Table 1).

**iPSC culture and organoid generation**

Human induced pluripotent stem cells were obtained from Gibco (Thermo) and cultured on GelTrex (Thermo) coated 6-well plates in StemFlex medium (Thermo). Cells were passaged using an EDTA dissociation method, and routinely karyotyped as described [30]. Vascular organoid generation was performed in a manner similar to that previously described [31]. Briefly, cells were dissociated and re-plated on Ultra Low Attachment 6-well plates (Corning) in StemFlex supplemented with RevitaCell (Thermo) overnight before commencement of differentiation protocol. On day 0 (d0), cells were collected from Ultra Low Attachment plates and spun down at 500xg before resuspension in Phase I media, which was comprised of APEL2 (Stem Cell Technologies) media supplemented with CHIR90921 (12 µM), and BMP4 (Thermo), FGF2, and VEGFA at 50 ng/ml (Stem Cell Technologies). Cells were incubated at 37 °C and 5 % CO2 for 3 days, before pelleting by gravitation and resuspension in Phase II medium. Phase II medium was composed of APEL2 medium, VEGFA at 100ng/ml, and FGF2 at 50ng/ml. On day 5 of the protocol, mesodermal blasts were embedded in a mixed matrix hydrogel composed of 60 % Collagen Type I (VitroCol – Advanced Biomatrix) and reduced Growth Factor Matrigel (Corning), and incubated in phase II medium supplemented with 15 % FBS. Fresh media was added on day 8, and sprouted cultures were isolated from the hydrogel at day 10 by scraping and centrifugation. Collected organoids were then cultured individually in 96 well Ultra Low attachment plates (Corning) before treatment at day 15.

**Treatment of vascular organoids with SARS-CoV-2 virus or antigens**

SARS-CoV-2 England 2 virus (Wuhan) was a kind gift from Christine Bruce, PHE. Recombinant trimeric spike glycoprotein (S) was produced in expressed in human embryonic kidney (HEK) 293F cells as previously described[32]. Nucleocapsid (N) protein was produced in *E.coli* bacteria and purified as described[33]. The levels of endotoxin in the N protein preparation was lower than 0.005 EU/µg. Vascular organoids were treated for 48 h with SARS-CoV-2 virus (M.O.I = 0.5) (n=3, 5-15 organoids per experiment). S and N (100 nM) were added to vascular organoids for 72 h in the presence and absence of IL-1β (20 ng/ml) (Peprotech) (n=5, 5-15 organoids per experiment).

## ELISA

Soluble IL6 (IL6) (Peprotech) and IL8 (Peprotech) were measured in the supernatant of untreated or treated organoids by ELISA. The levels of von Willebrand factor (VWF) were measured using polyclonal antibodies against VWF (Agilent). Plasma-derived VWF (Wilfactin) was used as a standard.

## qRT-PCR

Whole organoids were processed using the Micro RNEasy Kit (Qiagen, Germany) according to the manufacturer's instructions. Isolated RNA was quantified on the NanoDrop ND-100 (Thermo Scientific, USA) and cDNA was prepared using 1 µg RNA using the High Capacity cDNA Reverse Transcription Kit (Applied Biosystems, USA) according to the manufacturer's instructions using standard cycling conditions. cDNA was then diluted to 5ng before being combined with PowerUp SYBR Green Master Mix reagent (Applied Biosystems) and the relevant pre-designed PrimeTime IDT Primers. The absolute expression of the respective genes was calculated using the ΔCt method using *GAPDH* as an internal housekeeping control. Expression values were normalized to control conditions and log transformed before plotting using GraphPad Prism 7.

## Organoids and Lung sections staining

**Whole organoid staining.** Whole organoids from independent experiments were pooled, fixed for 15 min in PFA 4 %, washed in phosphate buffer saline (PBS)-Tween-20 (PBS-T 0.05 %) and incubated overnight in blocking buffer (2 % goat serum, 1 % bovine serum albumin (BSA)) supplemented with Triton-X100, and Sodium Deoxycholate. Samples were then incubated overnight at 4 °C in blocking buffer containing primary antibodies (Supplementary Table 2). Secondary Alexa Fluor antibodies (all from Invitrogen) were added for 2 h, washed before staining with DAPI. Upon labelling, samples were washed again in PBS-T, mounted in 0.5 % low melting point agarose (Fisher Scientific) in an Ibidi 8-well slide (Ibidi). Samples were then subject to serial dehydration in ethanol (30%, 50%, 70

%, 100 %) before clearing in ethyl cinnamate and imaging using Airyscan confocal microscopy.

**Organoid sections staining.** Vascular organoids from independent experiments were fixed for 15 min in PFA 4 % and frozen in optimum cutting temperature (OCT) compound (Tissue-Tek, The Netherlands). Sections (12 µm) were blocked with PBS containing 5 % BSA and 10 % goat serum for 1h and autofluorescence was quenched using ammonium chloride 50 mM for 20 min. Primary antibodies against ACE2 (ThermoFischer scientific) and CD144 (ThermoFischer scientific), Spike glycoprotein (Sinopharma), CD140b (Sigma-Aldrich), Ulex Europaeus Agglutinin I (UEA1), ACE2 (Thermofisher) and Biotinylated (VECTOR Laboratories) were incubated overnight at 4 °C. Click-iT™ TUNEL Alexa Fluor™ 647 Imaging Assay (ThermoFischer) was used to stain for apoptosis. Secondary antibodies were incubated for 1h at room temperature. Anti-NG2 Alexa Fluor 488 was incubated for 1h at RT (Supplementary Table 2). Slides are mounted using ProLong Gold Antifade Mountant with DAPI (Life Technologies). Sections were imaged using Epi fluorescent microscopy or Airyscan confocal microscope and analyzed using ZEN software and image J.

**Lung sections staining.** For paraffin sections, following rehydration and antigen retrieval, lung sections were treated with $H_2O_2$ 3 % for 15 min and blocked with PBS containing 5 % bovine serum albumin and 10 % goat serum for 1 h. Antibodies against Neural/glial antigen 2 (NG2), spike glycoprotein, Intercellular Adhesion Molecule 1, CD144 (VE-cadherin), VWF, platelet CD42b and fibrin (Supplementary Figure 2) were incubated overnight at 4 °C. Secondary antibodies were added for 1 h at room temperature. Nuclei were stained using DAPI. Lung autofluorescence was quenched using commercial kit (Vector laboratories) and slides mounted using ProLong Gold Antifade Mountant (Life Technologies). Sections were imaged using Epi fluorescent microscopy or Zeiss Axio Scan.Z1 microscope and analyzed using ZEN software and image J.

**Flow cytometry**

Organoids were harvested and dissociated using 200 U/ml collagenase type-II (Worthington) resuspended in HBSS solution (Sigma-Aldrich). For dissociation, cells were first washed twice with PBS before resuspension in collagenase solution and incubation at 37 °C for two 3 min intervals, with brief trituration between each step. The cells were blocked with PBS-FBS 10% for 20 min on ice. Dead/live cells were detected using Live/Dead Fixable Aqua Dead Cell Stain (Thermofischer). Cells were stained with anti

CD144-PEcy7, anti podoplanin-FITC, anti CD140b-PE, anti CD31-APC-Cy7 (all from Biolegend), anti ICAM-1-Biotin followed by Streptavidin PE-CF594 (BD), anti NG2-APC (Bio-Techne Ltd) for 20 min on ice. Cells were fixed and acquired by flow cytometry (Cyan, Beckman Coulter). Cell death is shown as the fraction of cells negative for Live/Dead Fixable Aqua Dead Cell Stain (live cells) over total cells detected within the endothelial fraction (CD31+), fibroblast ($CD140b^{+}NG2^{-}$) or pericytes ($CD140b^{+}NG2^{+}$) populations. Endothelial cells were defined as $CD31^{+}$ and $CD144^{+}$ double positive cells. The CD140b positive population contained both pericytes ($CD140b^{+}$ $NG2^{+}$) and fibroblasts ($CD140b^{+}$ $NG2^{-}$) as assessed by flow cytometry (Supplementary Figure 1). CD144 expression is assessed on $CD31^{+}$ cells (Supplementary Figure 1). Podoplanin and ICAM-1 expression are assessed on pericytes ($CD140b^{+}$ $NG2^{+}$) and fibroblasts ($CD140b^{+}$ $NG2^{-}$).

**Image analysis**

Image analysis was performed using Fiji [34]. For measures of VE-Cadherin in patient samples, 8-10 images across different slides and sections from each patient were taken and analyzed. Within each image, an ROI was drawn around a vessel and the mean intensity of the channel of interest was measured and plotted. For co-localization studies, ROIs were drawn around 5 distinct separate sectioned organoids and the Coloc2 method was applied to find the Pearson's Correlation Co-efficient.

**Data analysis**

All data were presented as means ± standard deviation (s.d). The significant difference groups were analyzed using either a One-Way ANOVA with multiple comparisons or a Kruskal-Wallis Test with multiple comparisons as indicated in figure legends using Prism 7 (GraphPad Software Inc, USA).

## Results

**Spike glycoprotein is detected in $NG2^{+}$-pericytes in autopsies of patients with COVID-19 and is associated with decreased endothelial permeability and thrombosis**

In the presence of conflicting data supporting endothelial viral uptake and with evidence of an emerging role of brain and heart pericytes in SARS-CoV-2 infection [19,20], we reasoned that SARS-CoV-2 targets pericytes in the microvasculature of the lung in patients with severe COVID-19, altering the crosstalk with endothelial cells and promoting vascular permeability and damage. As a first step, we assessed the presence of the spike glycoprotein in pericytes of lung tissues obtained *post mortem* from eight patients who died from COVID-19 (Supplementary Table 1). Immunostaining analysis of the viral spike glycoprotein revealed positive staining in NG2-positive ($NG2^{+}$) pericytes in 5 patients with COVID-19 while it was undetectable in age-matched non-COVID19 control lung autopsies (Figure 1A, B, C, Supplementary Figure 2). Notably, the presence of the spike glycoprotein in the lungs and its colocalization with pericytes was heterogenous among patients (Figure 1B, Supplementary Figure 2). $NG2^{+}$ $Spike^{+}$ cells were not lining the endothelium, in particular cells with high spike staining (as indicated in Figure 1B), and Spike signal was associated with ICAM-1 staining (Figure 1A, C), suggesting pericyte activation and detachment following infection.

Given that microvascular pericyte activation and apoptosis regulate microvascular endothelial permeability [35], we assessed the expression of the endothelial junctional protein VE-cadherin (CD144), as a marker for vascular permeability. In non-ventilated COVID-19 patients, CD144 expression on the microvascular vessels was decreased in SARS-CoV-2 infected lungs compared to control, most markedly in patients with confirmed macro and micro-thrombosis (Figure 1D, E, Supplementary Figure 3). These results show that spike glycoprotein strongly localizes with lung pericytes and this is associated with increased pericyte detachment and a decrease in endothelial barrier function, in particular in patients with thrombotic complications.

**Increased VWF and fibrin deposition in COVID-19 lungs patients with thrombotic complications**

CD144 is crucial for endothelial stability and blockade of CD144 contributes to coagulopathy, particularly in the lung microvasculature [36, 37]. We therefore assessed CD144 expression and markers of thrombosis in the lung autopsies of patients with COVID-19. Diffuse alveolar damage with extensive hyaline membrane rich in fibrin was observed in all patients (Figure 2A). Compared to lung autopsies from age-matched controls, we observed a significant decrease in CD144 expression (Figure 1D, E, 2B-D, Supplementary Figure 3) associated with platelet recruitment and VWF deposition in lung capillaries with low fibrin content (Figure 2B-D, Supplementary Figure 4). Thrombi in the large vessels were rich in VWF and fibrin, but with low content in platelets [38]. Megakaryocytes ($CD42b^{+}$) were also observed in some patients (Figure 2C). VWF was significantly higher in patients with thrombotic complications, supporting a key role for endothelial activation in thrombosis.

In order to assess whether CD144 downregulation is observed in other coronavirus family member viruses, we also assessed the expression of CD144, platelets ($CD42b^+$) and fibrin in lung autopsies from cases of Middle East respiratory syndrome coronavirus (MERS-CoV) and rhinovirus. A decrease in CD144 expression and an increase in fibrin deposition and platelet recruitment in the microvasculature was observed in MERS-CoV-infected lung compared to control lung tissue (Figure 2E) whereas CD144 expression was more evident in rhinovirus-infected lung without significant fibrin deposition or platelet recruitment (Figure 2F). These results suggest that in this small cohort of patients a decrease in CD144 expression and an increase in VWF deposition are observed in lung autopsies from patient with COVID-19, in particular with documented thrombosis.

**Preferential SARS-CoV-2 uptake by pericytes in a vascular organoid model**

In order to assess the direct role of SARS-CoV-2 on pericytes and endothelial cells, we generated 3D human vascular organoids [30, 31] (Figure 3A) comprised of vascular endothelium ($CD144^+$), and pericytes ($CD140b^+$) (Figure 3B, Supplementary Figure 1). The percentage of endothelial cells varies between 20-50% endothelial cells, 20-50% for $NG2^+CD140b^+$ and 10-40% $NG2^-CD140b^+$. Co-staining of vascular organoid sections with ACE2 and CD144 antibodies indicated a distribution of ACE2 outside the branching junctions of endothelial cells (Figure 3C). ACE2 strongly colocalized with pericytes ($NG2^{+,}$ $CD140b^+$) with lower colocalization with the endothelial marker UAE1 (Figure 3D-F). Organoids were then exposed to live SARS-CoV-2 virus (M.O.I 0.5) for 48 h and the distribution of the spike glycoprotein assessed using immunofluorescence imaging (Figure 3G). Colocalization analysis showed a strong presence of the spike in $CD140b/NG2^+$ (Figure 3H) with lower levels detected in UAE1-positive endothelial cells (Figure 3H). SARS-CoV-2 infection of vascular organoid was associated with increased cell apoptosis as assessed using TUNEL staining (Figure 3I, J). TUNEL staining was observed in both UAE1-positive endothelial cells and pericytes ($CD140b/NG2^+$), however it was significantly higher in pericytes compared to endothelial cells (Figure 3K). SARS-CoV-2 infection progressively altered the expression and distribution of NG2 within the infected organoids associated with a decrease in CD144 expression (Figure 3L-N). The decrease in CD144 was not due to the loss of endothelial cells as shown using UAE1 staining (Supplementary

Figure 5). These results show that infection of vascular organoids with SARS-CoV-2 induces pericyte apoptosis and decreases CD144 expression through a preferential uptake of the virus by pericytes. This is associated with a disruption of the vessel architecture, detachment of pericytes and loss of vascular integrity.

**Spike glycoprotein and nucleocapsid protein impair endothelial integrity and survival without significant activation**

In order to assess whether the spike glycoprotein is responsible for altered endothelial integrity and cell survival, vascular organoids were treated with i) recombinant active trimeric spike glycoprotein (S) (100 nM) [32, 39]; ii) nucleocapsid protein (N) (100 nM) [33] or iii) a combination of both (S + N) for 72 h. Endothelial survival was assessed using live/dead fixable dye staining, while endothelial cell activation and permeability was assessed using ICAM-1 and CD144 expression, respectively (Supplementary Figure 1). Treatment of vascular organoids with S, N or S+N decreased endothelial cell survival as assessed by a decrease in the fraction of live endothelial cells from total cells (Figure 4A, B). Co-stimulation with IL-1β, a potent pro-inflammatory cytokine, did not alter cell death compared to viral antigen alone. Similar to SARS-CoV-2 infection, treatment of vascular organoids with viral antigens decreased the number of endothelial cells ($CD144^+$/$CD31^+$ cells; Figure 4C). Treatment with viral antigens did not induce endothelial cell activation as assessed by the expression of ICAM-1 on endothelial cells (Figure 4D). No significant change in the levels of IL6, IL8 and VWF in the supernatants were observed following viral antigen treatment compared to untreated organoids (Figure 4E, F, G). These results support a role for N and S protein in regulating endothelial cell death and integrity without inducing significant endothelial activation.

**Spike glycoprotein and nucleocapsid protein induce pericyte death in vascular organoids**

We further assessed pericyte and fibroblast survival and activation following viral antigen treatment by flow cytometry. Viral antigens N and S decreased pericyte survival as assessed by a decrease in the live fraction of pericytes among total cells (Figure 4H, I). Despite the presence of ICAM-1-positive pericytes in patient lung sections, no significant activation of pericytes was observed in vascular organoids as measured by the expression of ICAM-1 on $CD140b^+NG2^+$ pericytes (Figure 4J). Addition of IL-1β did not alter cell survival or activation compared to antigen alone (Figure 4J). No significant cell death of activation was observed in the fibroblast population (Supplementary Figure 6). These results show that S and N antigens alter pericyte survival without inducing direct activation.

**Spike and N proteins alter gene expression linked to vascular integrity and permeability in vascular organoids**

To investigate the mechanisms by which viral infection of the vasculature drives the loss of CD144 expression, we treated vascular organoids with S and N for 4, 24 and 72 h hours to assess transcriptional changes in key genes involved in the maintenance of vascular barriers by qRT-PCR. These included genes encoding for angiopoietin-1 (*ANGPT1*), angiopoietin-2 (*ANGPT2*), sphingosine kinase 1 (*SPHK1*), sphingosine kinase 2 (*SPHK2*), Notch receptor 3 (*NOTCH3*), beta catenin 1 (*CTNNB1*), Jagged Canonical Notch Ligand 1 (*JAG1*), Jagged Canonical Notch Ligand 2 (*JAG2*), CD144 (*CDH5*), CD31 (*PECAM*), Tie2 (*TEK*), *ACE2*, *PDGFRB*, Toll like receptor 4 (*TLR4*), and Integrin alpha 4 (*ITGA4*). Compared to untreated organoids, we found significant down-regulation in *NOTCH3*, *SPHK2*, and *TEK*, while in contrast we saw a marked upregulation of *ACE2*, *ANGPT2* and *SPHK1* (Figure 3K, Supplementary Figure 7). No significant changes in *CDH5*, *ANGPT1* or other genes was observed. These results show that S and N treatments alter the expression of gene regulating vascular integrity and permeability.

## Discussion

In this study, we used lung autopsy sections from patients with COVID-19 and a 3D vascular organoid model to show that SARS-CoV-2 and its antigens S and N decrease the expression of the adhesion junction molecule CD144 and alter endothelial cell and pericyte survival without inducing endothelial activation. We also observe a decrease in CD144 expression in the lung microvasculature of COVID-19 patients that is associated with increased platelet recruitment and VWF deposition. The preferential uptake of SARS-CoV-2 by pericytes in the vascular organoid alters the expression of pericyte and endothelial genes regulating endothelial permeability and integrity, increasing cell permeability and death.

During homeostasis, the integrity of the vasculature is maintained through a tight crosstalk between mural and endothelial cells. Pericytes and endothelial cells establish connections to maintain endothelial quiescence through cell-cell contact via “Peg-and-socket” like membrane structures which include tight (claudin, occludin and JAM-1), gap (connexin43) and adherent (N-cadherin) junctions and through focal adhesion plaques allowing indirect interactions between both cells via the extracellular matrix through integrins [40]. Pericyte and endothelial cells can also communicate through paracrine signaling of growth factor (angiopoetin-1 and PDGF), their receptors (Tie2 and PDGFRb) and juxtacrine signaling (Jagged1-Notch3) [6]. Following inflammatory or infectious challenge, as observed during severe SARS-CoV-2 infection, the protective effect of the endothelium is lost, promoting a thrombotic and inflammatory microenvironment, supporting platelet recruitment, activation of the coagulation cascade and thrombosis. Indeed, endotheliitis, endothelial dysfunction and death are hallmarks of severe SARS-CoV-2 infection [1, 41]. Using a vascular organoid model, we observed that SARS-CoV-2 or viral antigens S and N downregulate CD144 expression on endothelial cells, increasing cell death. CD144 mediates endothelial cell interactions and antibodies blocking this interaction increase endothelial permeability, apoptosis, vascular instability and hemorrhages [42, 43]. This can also expose the prothrombotic extracellular matrix to blood cells, such as platelets, promoting a thrombotic state [37]. The detection of spike glycoprotein in pericytes in vascular organoids and COVID-19 lung autopsies suggests preferential uptake of SARS-CoV-2 by pericytes, probably due to the high expression of ACE2, is a key contributor for pericyte and endothelial damage [44,45]. This effect was mediated by pericyte infection, as the presence of the spike glycoprotein was mainly observed in pericytes, both in vascular organoids and COVID-19 lung autopsies. The increase in apoptosis observed could be due to a reduction in the capacity of infected pericytes to support an endothelial network. This, in turn, produces pro-apoptotic factors resulting in EC death as recently shown using primary cardiac pericytes [22]. Using a cortical organoid model, pericytes were shown to serve as a replication site for SARS-CoV-2 allowing viral production, transport and infection of other cells such as astrocytes mediating their death [23]. In a model of vascular organoids, SARS-CoV-2 infection and replication was inhibited by human soluble recombinant ACE2, although the cell type involved were not identified[29]. In this study, we identified the pericytes as the main cells responsible for SARS-CoV-2 uptake within the vascular organoids. SARS-CoV-2 infection of pericytes in vascular organoids results in increased apoptosis in both pericytes and endothelial cells, thereby increasing vascular permeability. Moreover, the loss of pericytes increases endothelial cell sprouting and intussusceptive angiogenesis in SARS-CoV-2-infected lungs combined with disruption of intercellular junctions, cell swelling and a loss of contact with the basal membrane [5]. Despite a lower expression of ACE2 on endothelial cells, SARS-CoV-2 can also directly impair endothelial permeability by acting directly on endothelial cells[46]. The effect of spike protein mediated endothelial dysfunction was further increased on diabetic endothelial cells, supporting a role for chronic disease-induced vessel damage in the high susceptibility to cell damage. The effect of the spike is not limited to CD144, as a significant decrease in the tight junction protein JAM-A and the gap junction protein connexin-43 were also observed [46]. We did not observe an alteration in the gene expression of CDH5, suggesting CD144 undergoes internalization and degradation. Indeed, it was recently shown that the spike protein-induced internalization of ACE2 triggered the subsequent internalization and degradation of key junction proteins impairing endothelial barrier integrity [45].

In addition to the decrease in endothelial junction protein CD144, we observed that addition of the spike and N proteins induced gene expression of angiopoietin-2 while downregulating NOTCH3. These data are consistent with previous observations showing that knockdown of NOTCH3 using siRNA increases proinflammatory genes including angiopoietin-2 and ICAM-1, induces pericyte dysfunction and pericyte-endothelial cell interaction destabilization promoting vascular leakage.  Angiopoietin-2 is a non-signal transducing ligand of Tie-2 produced by endothelial cells. Activation of the Tie-2 signaling pathway by angoipoietin-2 destabilizing the vasculature by inhibiting the protecting effect of angiopoietin-1. Moreover, angiopoietin-2 induces pericyte damage and detachment, increasing vascular leakage and immune cell migration. This process increases plasminogen activator inhibitor-1 (PAI-1) in endothelial cells, which supports coagulation, endothelial activation and inflammation [47]. An increase in the levels of angiopoietin-2 [48] and PAI-1 [49] is observed in patients with COVID-19 and associates with thrombo-embolic events.

Pericyte activation and dysfunction increase EC procoagulant activity and endothelial permeability, as constitutive hypoplasia of pericytes increases VWF release, tissue factor expression and platelet adhesion. In this study, we did not observe a significant increase in VWF in the supernatant of vascular organoids treated with viral antigens, suggesting that VWF release associated with endothelial activation may result from the dysregulated immune/inflammatory response associated with SARS-CoV-2 infection rather than pericyte or endothelial cell infection [50-54]. With the lack of efficacy of anti-platelet drugs in improving outcome in severe COVID-19 patients [38, 55, 56] endothelial activation and release of VWF represents a potential target to limit thrombosis in COVID-19 patients. Recently, platelet activation and secretion of S100 A8/A9, a damage-associated molecular pattern, was shown to induce endothelial activation, supporting a role for platelet recruitment and activation in endotheliitis [11]. These effects can be exacerbated by comorbidities, which can increase endothelial and pericyte responses to infection. Further studies using vascular organoid models mimicking disease state can shed light on the mechanisms of endothelial activation during SARS-CoV-2 infection.

Our study has however some limitations. The detection of spike glycoprotein in lung pericytes was heterogenous in our small cohort of lung autopsies. This could be due to different infection rate and incubation time, patients' comorbidities or other unknown factors. The infection of pericytes was shown using our vascular organoid model as well as a cortical organoid model, supporting a key role for pericyte infection in altering the communication with neighboring cells. Moreover, these observations need to be validated in a larger number of lung autopsies, due to the limited sections available for this study.

In conclusion, we propose that direct infection of pericytes by SARS-CoV-2 induces pericyte damage and dysregulates the crosstalk with endothelial cells, increasing vascular permeability. However, endothelial activation is more likely regulated by the inflammatory response and platelet recruitment and activation in particular in the microcirculation rather than direct viral infection and combined endothelial activation and increased vascular permeability promotes vasculopathies.

## Declarations

## Funding

AOK is a Henry Wellcome fellow (218649/Z/19/Z). JR is a British Heart Foundation Intermediate Fellow (FS/IBSRF/20/25039). This research was supported by BHF Accelerator Awards to JR and AOK (AA/18/2/34218). This research was funded, in whole or in part, by the Wellcome Trust (218649/Z/19/Z), British Heart Foundation (AA/18/2/34218) and COMPARE. A CC BY or equivalent licence is applied to AAM arising from this submission, in accordance with the grant's open access conditions. M.C. was supported by the University of Southampton Coronavirus Response Fund. M.C. was also supported by the International AIDS Vaccine Initiative (IAVI) through grant INV-008352/ OPP1153692 and the IAVI Neutralizing Antibody Center through the Collaboration for AIDS Vaccine Discovery grant INV-008813/OPP1196345, both funded by the Bill and Melinda Gates Foundation.

**Author contributions**

Contribution: AOK and JR designed research, performed research, analyzed data and wrote the paper; JSR performed research and analyzed data; JHB and MC contributed to data analysis; MLN, JDA, MC, AF contributed vital new reagents; EY, PGM, GT, AGR contributes to clinical information and samples collection; ZS, HH and MP contributed vital new reagents, performed experiments and analyzed data; All authors read and approved the paper.

**Conflict of interest**

The authors have no conflicts of interest to declare that are relevant to the content of this article.

**Data availability**

Any relevant data is available on request.

**Figure 1: Detection of the spike glycoprotein in lung pericytes and decreased CD144 expression in COVID-19 patients.** (A) Immunofluorescence imaging of $NG2^+$, spike glycoprotein and ICAM-1 in formalin fixed and paraffin embedded lung from patient who died from COVID-19 and control COVID-19$^-$ (Control) lung sections. (B) High magnification of immunofluorescence imaging of $NG2^+$ and spike glycoprotein in lung section. Yellow arrows indicating $NG2^+$ cells without Spike glycoprotein lining vessels, while red arrows indicate infected cells. (C) Quantification of ICAM-1 expression using ImageJ in lung autopsies from two patients who died from non-respiratory-associated diseases, 4 COVID-19 patients without thrombosis and 4 patients with detectable thrombosis (3-6 areas per slide). (D) Representative immunofluorescence imaging of CD144 (VE-cadherin) in lung sections. (E) Quantification of CD144 levels using ImageJ. One-Way ANOVA with multiple comparisons performed for each statistical test with significance at (*** p = < 0.001).

**Figure 2: Decreased CD144 expression is associated with increased VWF deposition in the lung of patients with COVID-19.** (A) H&E staining of lung sections from patients who died from COVID-19 with or without evidence of thrombosis. (B, C) Immunofluorescence imaging of CD144, VWF, platelets (CD42b) and fibrin in formalin-fixed and paraffin-embedded lung sections. (D) Quantification of VWF in lung sections. (E, F) Staining of CD144, CD42b, fibrin and DAPI in lung sections from patients infected with (E) MERS-CoV and (F) Rhinovirus. Images were captured using a Zeiss Observer 7 Epifluorescence microscope and slide scanner Axio Scan Z1. (Kruskal Wallis Test performed on a total of 3-5 lung sections from 3 control patients, 3 COVID-19 patients with thrombosis and 3 without evidences of thrombosis (* p = < 0.05, ** p = < 0.01, *** p = < 0.001, **** p = < 0.0001).

**Figure 3: Pericyte preferential uptake of SARS-CoV-2 increases vascular permeability and endothelial and pericyte death in vascular organoids.** (A) Vascular organoids were generated using a step wise differentiation of human induced pluripotent stem cells. (B) Cell composition and distribution in mature vascular organoids were assessed using immunofluorescence whole mount microscopy. (C) Immunofluorescence imaging of ACE2 and CD144 positive cells in organoids (12 µm organoid sections. (D) Sections of vascular organoids were co-stained for pericytes (NG2) and ACE2. (E) Staining for UAE-1, ACE2, CD140b and DAPI in vascular organoids. (F) Co-localization analysis between ACE2 and CD140b compared ACE2 co-localization with UAE1 as measured by the Pearson's Correlation Co-Efficient. (G) Co-staining for the spike glycoprotein in endothelial organoids with $CD140b^+$ cells treated with SARS-CoV-2. (H) Quantification of co-localization of the spike glycoprotein between endothelium and pericytes. (I) Sections of vascular organoids were stained with apoptosis marker TUNEL and nuclei staining DAPI. (J) Quantification of TUNEL staining in control and SARS-CoV-2 treated vascular organoids. (K) Co-localization of the TUNEL staining with UAE-1 (endothelium)and pericyte ($CD140b^+$). (L) Staining of control and SARS-CoV-2 infected organoids for CD144, pericytes (NG2) and nuclei (DAPI). (M) Immunofluorescence imaging of CD144 and nuclei (DAPI) in whole vascular organoids treated with SARS-CoV-2 (M.O.I= 0.5) for 48 h. (N) Quantification of CD144 staining in control and SARS-CoV-2 treated vascular organoids. (O) Staining for NG2, UAE-1, TUNEL, and spike in control and SARS-CoV-2 infected organoids (48h). N = 4 for organoids experiments, where one replicate is a total of 8-10 individual organoids pooled for assays from each of 4 independent biological replicates. Each biological repeat was a separate differentiation protocol followed by viral infection. For co-localizations un-paired T-Tests were performed (** p = < 0.01) Schematic created on Biorender.com.

**Figure 4: Recombinant viral antigens S and N increase vascular permeability and death without endothelial activation.** (A-D) Vascular organoids were treated with spike glycoprotein (S) (100 nM), nucleocapsid (N) or a combination of both proteins for 72 h in the presence or absence of IL1-β (20 ng/ml) compared to control was measured by flow cytometry. (A) Representative flow cytometry blots of endothelial cell treated with S, N, and S and N antigens in the presence and absence of IL1-β. (B) Fold change in the live population of $CD31^+$ cells from the total population using Live/Dead fixable dead cell stain. (C) Fold change in CD144 expression on $CD31^+$cells compared to untreated organoids and (D) fold change in ICAM-1 expression on $CD31^+$ cells in treated organoids versus untreated were measured by flow cytometry. (E) Detection of the levels of soluble IL6, (F) IL8 and (G) VWF in the supernatant of organoids treated for 72 h with viral antigens in the presence or absence of IL-1β by ELISA (n=3 independent experiments, 3-4 replicate per experiment). (H) Representative flow cytometry plots of pericytes treated with S, N, and S and N antigens in the presence and absence of IL1-β (20 ng/ml). (I) Live/dead $NG2^+$ cells from the total population, (J) fold change in ICAM-1 expression on $CD140^+NG2^+$ cells in treated organoids versus untreated were measured by flow cytometry. (K) qPCR of vascular organoids treated with combination S and N proteins for 0, 4, 24 and 72 h for genes regulating endothelial permeability. *N* = 3 for experiments, where one replicate is a total of 12-15 individual organoids pooled for assays from each of 3 independent biological replicates (independent differentiations). One-Way ANOVA with multiple comparisons performed for each statistical test with significance at (* p = < 0.05, ** p = < 0.01, *** p = < 0.001, **** p = < 0.0001).

**Figure 1**

1

2

**Figure 2**

**Figure 3**

**Figure 4**

## Supplementary Material

cvac097_Supplementary_Data

