# Supplementary material for: Preferential uptake of SARS-CoV-2 by pericytes potentiates vascular damage and permeability in an organoid model of the microvasculature
Source: Cardiovasc Res. 2022 Jun 16;118(15):3085–96. doi: 10.1093/cvr/cvac097 (PMC9214165; doi:10.1093/cvr/cvac097)
Supplement: cvac097_Supplementary_Data [file cvac097_supplementary_data.docx]

**SUPPLEMENTARY MATERIAL**

**Preferential uptake of SARS-CoV-2 by pericytes potentiates vascular damage and permeability in an organoid model of microvasculature**

Abdullah O. Khan*^^1^, Jasmeet S. Reyat^1^^, Harriet Hill^2^, Joshua H. Bourne^1^, Martina Colicchia^1^, Maddy L. Newby^3^, Joel D. Allen^3^, Max Crispin^3^, Esther Youd^4^, Paul G. Murray^2,5^, Graham Taylor^2^, Zania Stamataki^2^, Alex G. Richter^2^, Adam F. Cunningham^2^, Matthew Pugh^2^**,** Julie Rayes*^1^.

1. Institute of Cardiovascular Sciences, College of Medical and Dental Sciences, University of Birmingham, Vincent Drive, B15 2TT, Birmingham, U.K.

2. Institute of Immunology and Immunotherapy, University of Birmingham, Birmingham, B15 2TT, U.K.

3. School of Biological Sciences, University of Southampton, Southampton SO17 1BJ, U.K.

4. Forensic Medicine and Science, University of Glasgow, Glasgow, UK

5. Health Research Institute, University of Limerick, Limerick, Ireland

*Correspondence to [j.rayes@bham.ac.uk](mailto:j.rayes@bham.ac.uk) and [a.khan.4@bham.ac.uk](mailto:a.khan.4@bham.ac.uk). ^These authors have contributed equally.

**
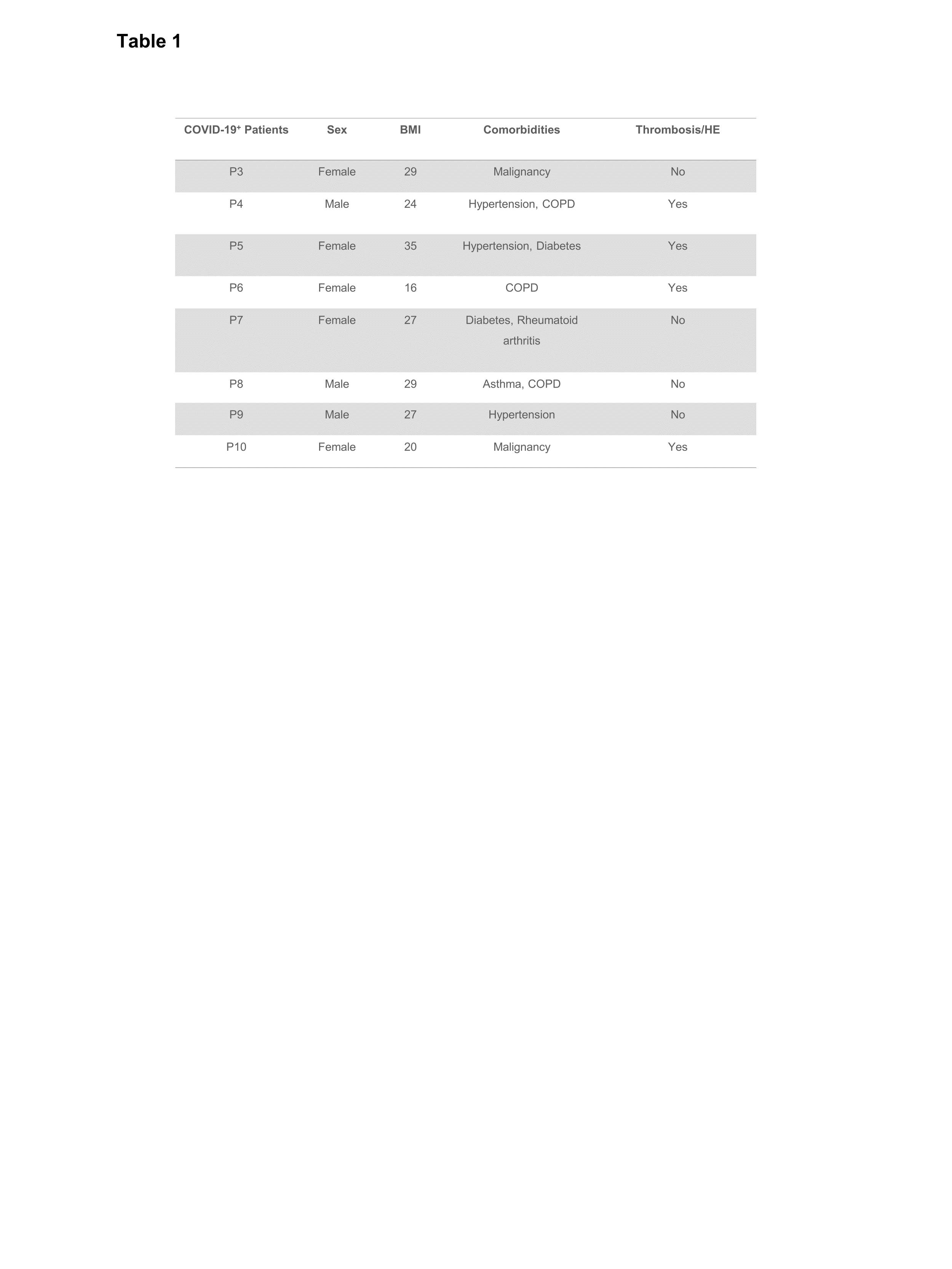
**

**Supplementary Table 1: Comorbidities of patients with COVID-19.**

| Primary antibodies | Source | Catalogue number | Final concentration (µg/ml) |
| --- | --- | --- | --- |
| VWF | DAKO | A0082 | 2 |
| CD144 | Invitrogen | 14-1449-82 | 5 |
| CD140b | Bio-RAD | 74603104 | 10 |
| CD140b | Sigma | SAB4700458 | 10 |
| Spike | Sino Biological | 40150-R007 | 10 |
| SARS-CoV-2 (S1) | Native Antigen | MAB12424 | 10 |
| NG2 | ThermoFischer | **MA1-90928** | 10 |
| ACE-2 | Invitrogen | PA520048 | 10 |
| ICAM-1  (CD54)-biotin | Biolegend | 228596 | 10 |
| Fibrin | EMD millipore | MAB522155 | 10 |
| CD42b | Abcam | Ab183345 | 10 |
| UAE-1 biotinylated | Vector labs | B-1065 | 10 |
| Anti NG2-Alexa Fluor 488 | EMO Millipore | AB5320A4 | 1:50 |

**Supplementary Table 2:** List of primary antibodies used for immunofluorescence staining.

**
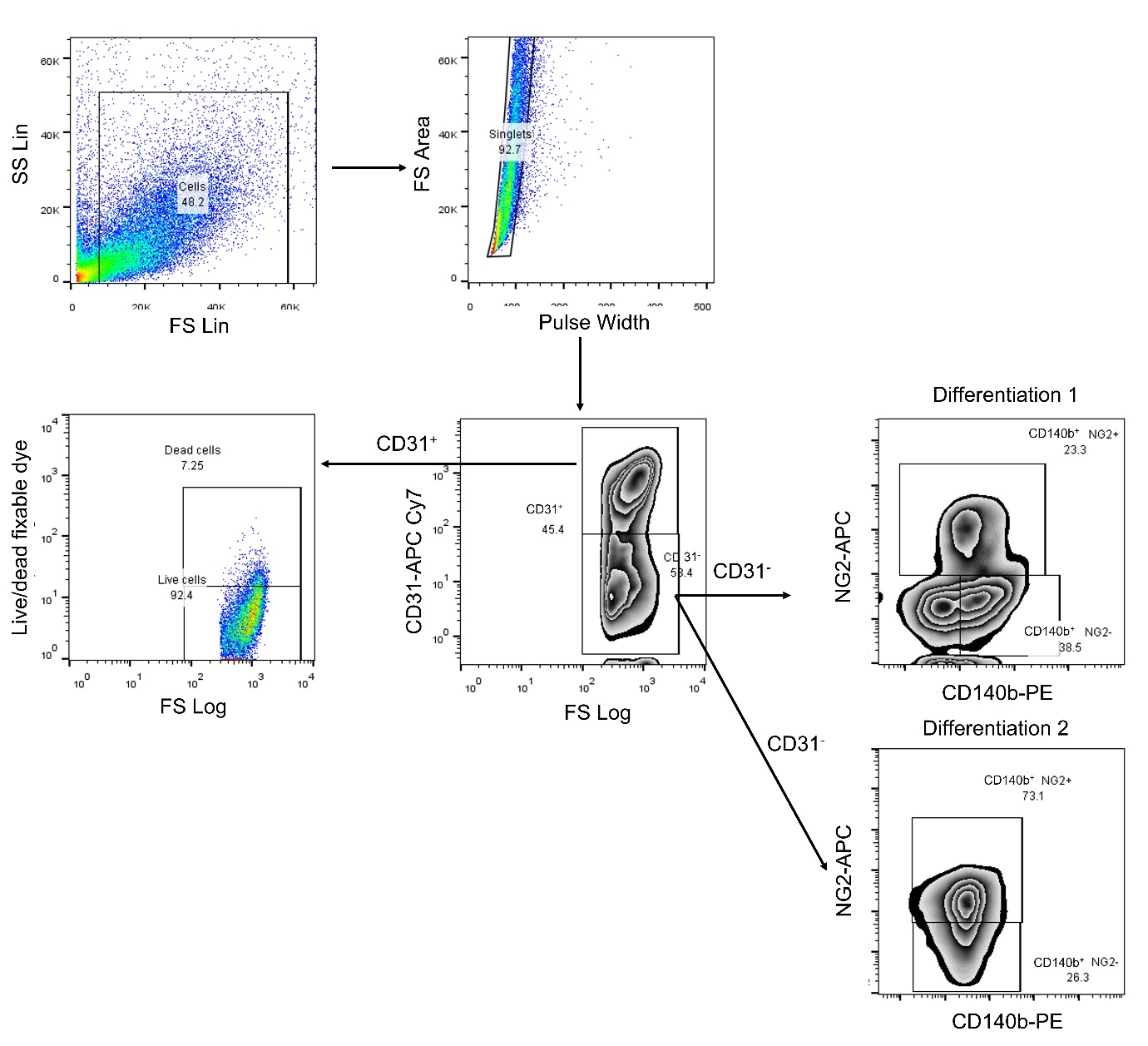
**

**Supplementary Figure 1:** **Flow cytometry gating strategy to identify different populations in collagenase-digested vascular organoids.** Gating strategy employed to determine viability in the endothelial, pericyte, and fibroblast compartments of antigen treated organoids. Plots show the variation in the percentage of endothelial cells (CD31^+^), pericytes (CD140b^+^NG2^+^) and fibroblast (CD140b^+^NG2^-^) from the total cells between differentiations.

**
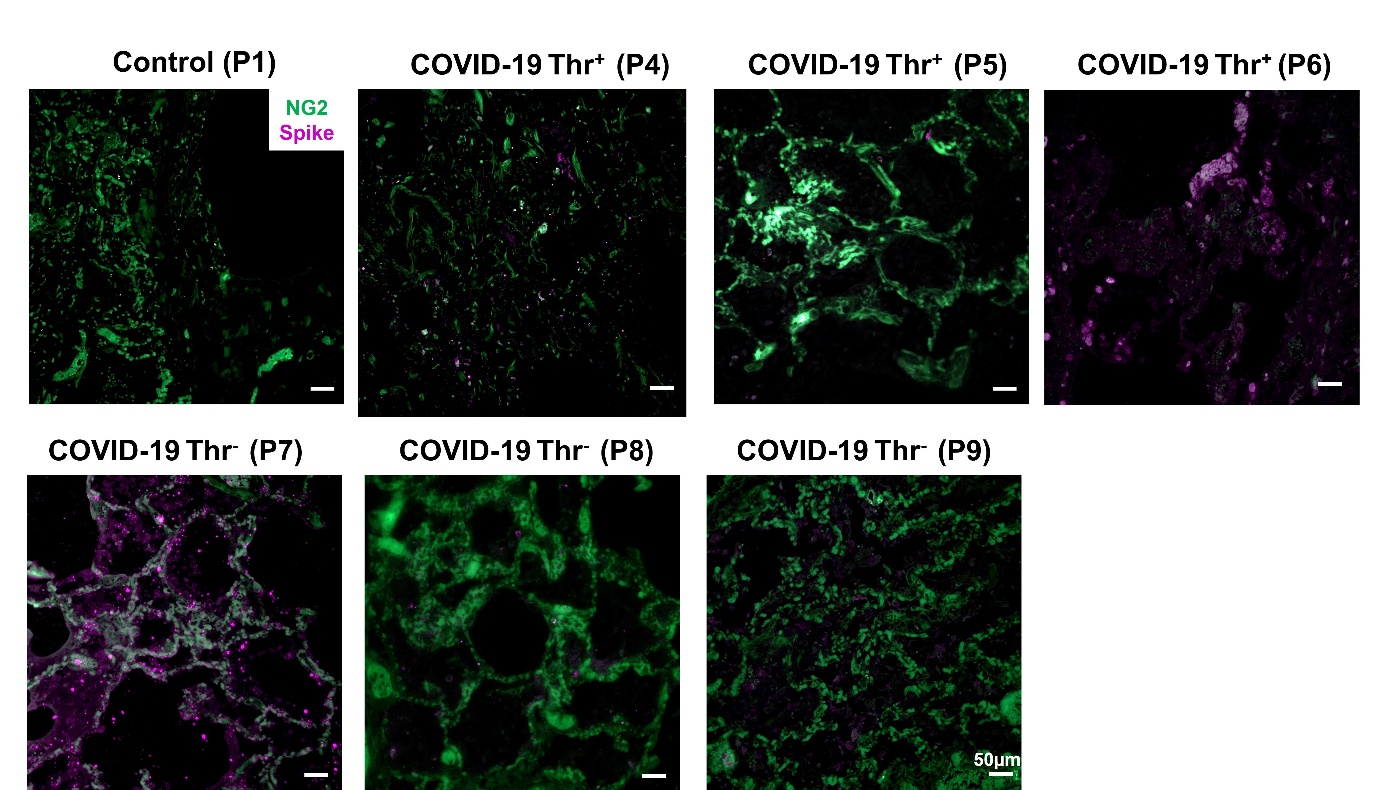
**

**Supplementary Figure 2: Heterogenous spike glycoprotein colocalization with lung pericytes in patients with COVID-19.** Representative immunofluorescence imaging of NG2 (pericytes) and spike glycoprotein in formalin-fixed and paraffin-embedded lung from patients who died from COVID-19 with and without evidence of thrombosis. Images were captured using Epi fluorescence microscope.


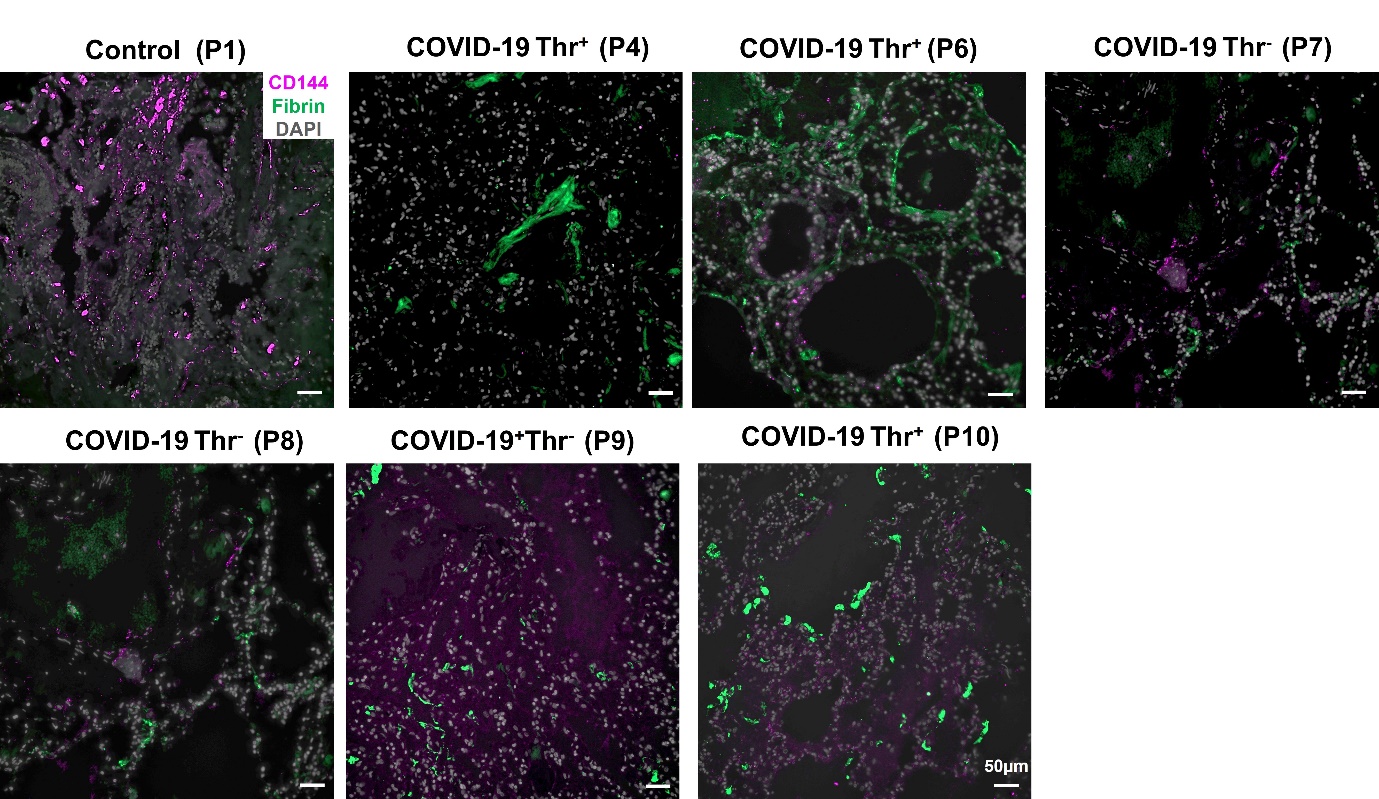


**Supplementary Figure 3: Decreased CD144 expression in the lung of COVID-19 patients.** Representative immunofluorescence imaging of CD144, fibrin and nuclei (DAPI) in formalin-fixed and paraffin-embedded lung from patients who died from COVID-19 with and without evidence of thrombosis. Images were captured using Epi fluorescence microscope.


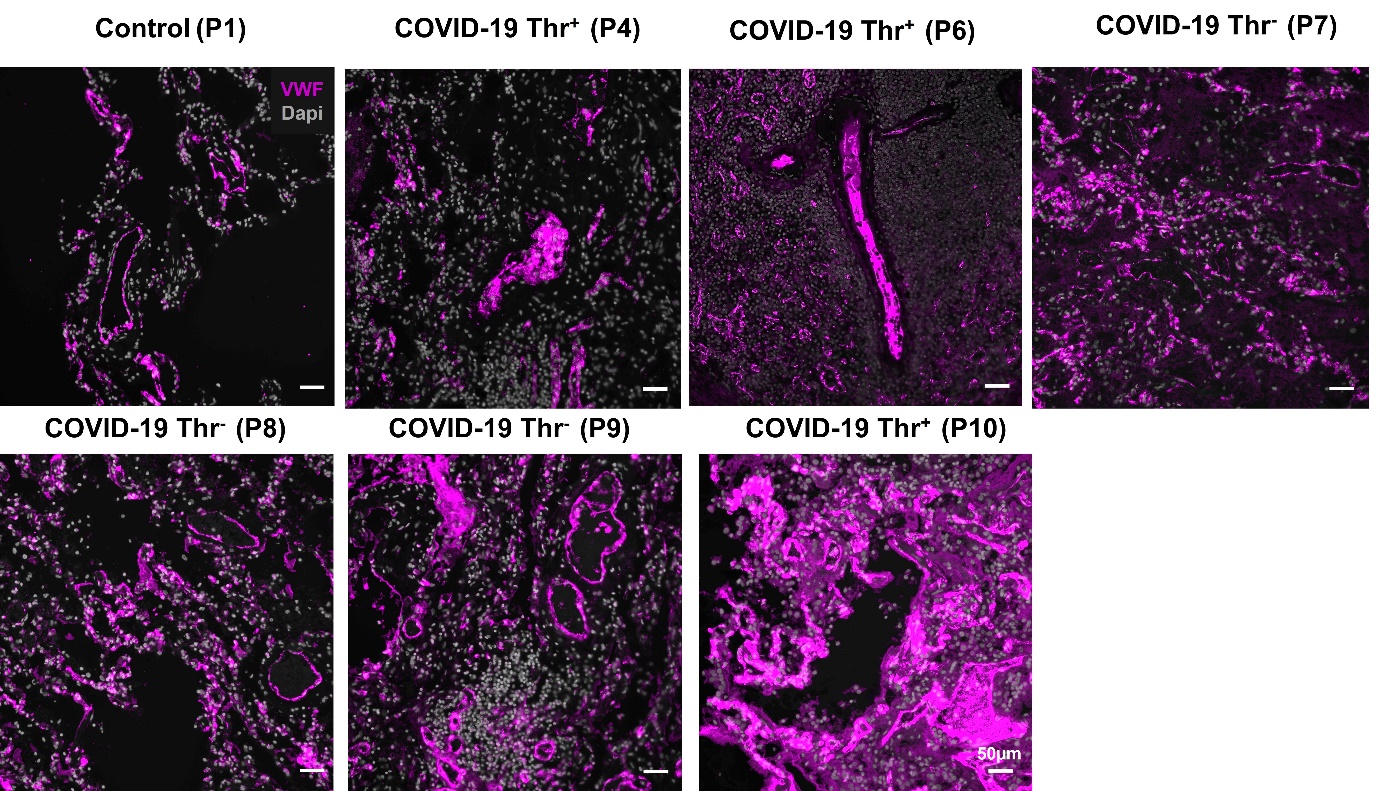


**Supplementary Figure 4: Increased VWF deposition in the lungs of patients with COVID-19.** Representative immunofluorescence imaging of VWF and nuclei (DAPI) in formalin-fixed and paraffin-embedded lung from patients who died from COVID-19 with and without evidence of thrombosis. Images were captured using Epi fluorescence microscope. Bar 50µm.


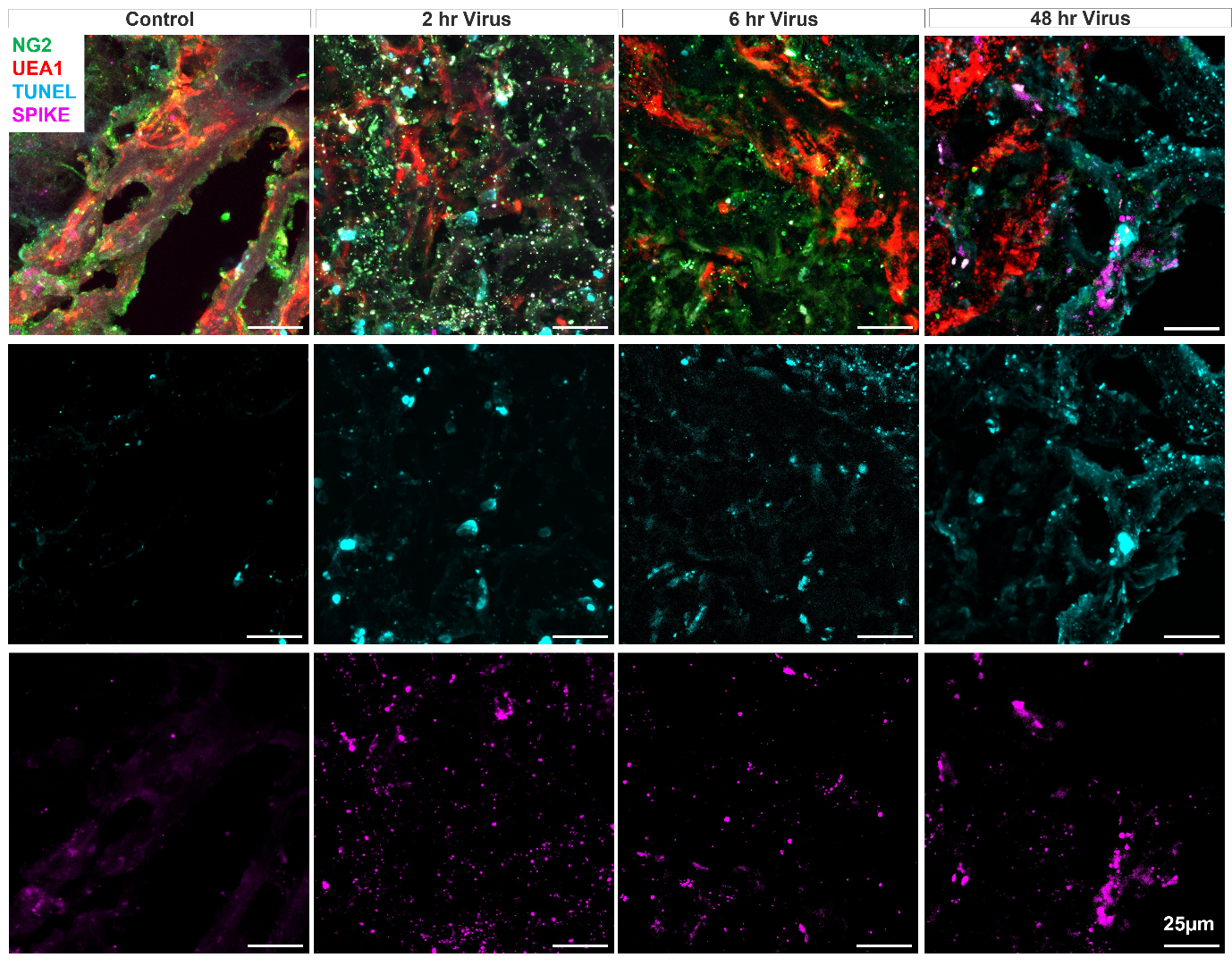


**Supplementary Figure 5:** **SARS-CoV-2 increases cell apoptosis in vascular organoids.** Immunofluorescence imaging of NG2, UAE-1, TUNEL and spike in control and SARS-CoV-2 infected organoids. Images were captured using Airyscan microscope.


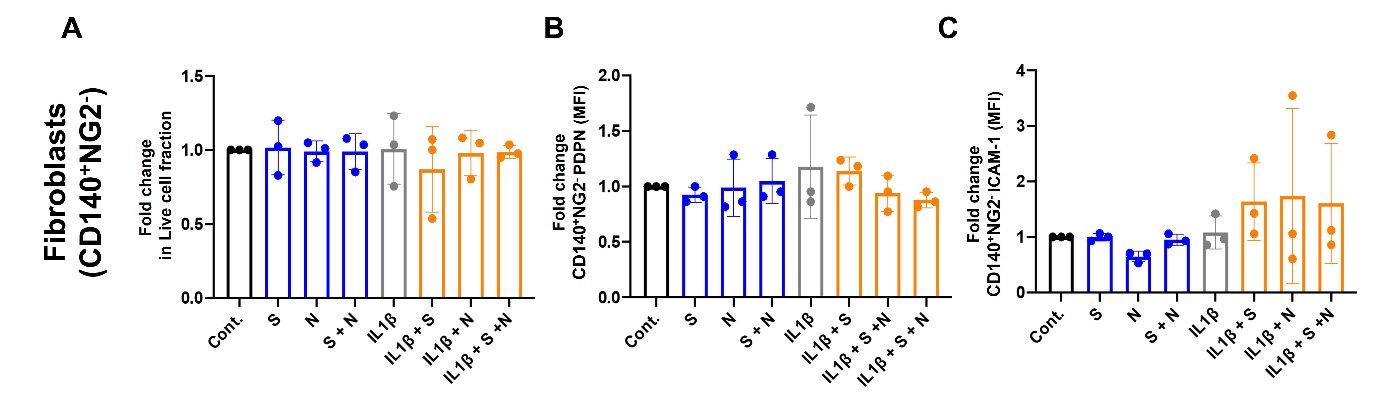


**Supplementary Figure 6: S and N proteins do not alter fibroblast survival or activation.** Podoplanin and ICAM-1 expression in the fibroblast population of vascular organoids treated with S, N and S+N in the presence or absence of IL-1β. (A) Live/dead CD140^+^NG2^-^cells from the total population, (B) Fold change in podoplanin expression and (C) ICAM-1 on CD140^+^NG2^-^ cells in treated organoids versus untreated were measured by flow cytometry.


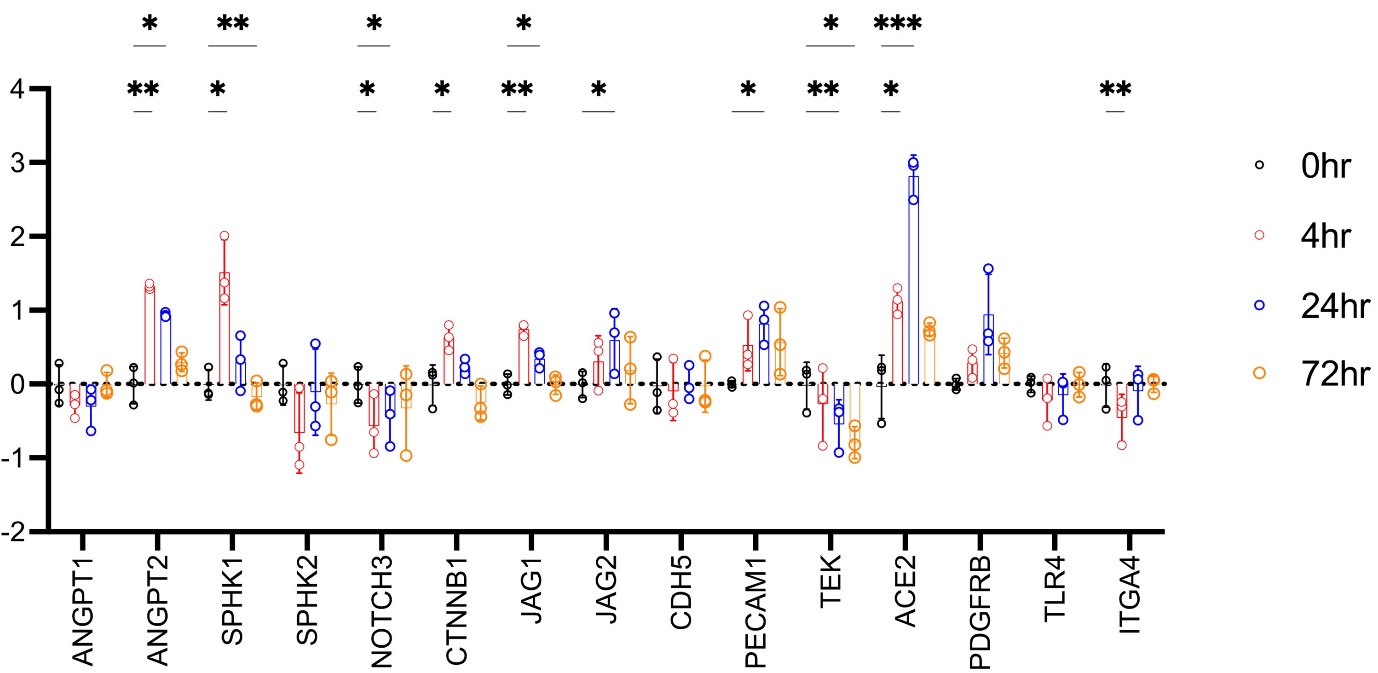


**Supplementary Figure 7: S and N proteins alter the expression of key genes regulating vascular permeability and integrity.** Individual values for heatmap plotted in figure 3K. 3 independent differentiations and treatments with SARS-CoV-2 Spike and nucleocapsid proteins were performed, with RNA extracted at 4, 24 and 72 hours post treatment. Individual values are plotted. (*N* = 3, standard deviation (S.D) plotted, two-way ANOVA with multiple comparisons, * p < 0.05, ** p < 0.01, *** p < 0.001).
